# Correlación entre número de cepillados por día y cpod en escolares de 12 años de la parroquia El Vecino (Cuenca, Ecuador) 2016

**DOI:** 10.21142/2523-2754-0901-2021-042

**Published:** 2021-03-11

**Authors:** Hannia Estefanía Fernández-Pesantez, Adriana Belén Romo-Cardoso, Gladys Eulalia Cabrera-Cabrera

**Affiliations:** 1 Carrera de Odontología, Universidad Católica de Cuenca. Cuenca, Ecuador. hanniafernandez31@gmail.com, aromoc@ucacue.edu.ec, gcabrera@ucacue.edu.ec Universidad Católica de Cuenca Carrera de Odontología Universidad Católica de Cuenca Cuenca Ecuador hanniafernandez31@gmail.com aromoc@ucacue.edu.ec gcabrera@ucacue.edu.ec

**Keywords:** caries dental, índice CPOD, cepillado dental, dental caries, CPOD index, Brushing dental

## Abstract

**Objetivo:**

Correlacionar el número de cepillados por día y el índice CPOD en los escolares de 12 años de la parroquia El Vecino (Cuenca, Ecuador) en 2016.

**Materiales y métodos:**

El estudio fue de tipo comunicacional, cuantitativo, descriptivo y relacional. La población estuvo conformada por 279 fichas que pertenecen al estudio del mapa epidemiológico para los escolares de la parroquia El Vecino. La ficha de observación incluyó los siguientes datos: número de registro, edad, sexo, parroquia, índice de COPD.

**Resultados:**

En lo referente a la correlación entre el número de cepillado por día y el índice de CPOD, se demostró que existe una correlación inversa, la estadística significativa presentó un valor de p = 0,029.

**Conclusión:**

El presente estudio demostró que existe una correlación entre el número de cepillados por día y el índice CPOD.

## Abstract (en)

**Objective:**

Correlate the number of brushes per day and the decay-missing-filled teeth (DMFT) index in 12-year-old school children from the parish “El Vecino Cuenca - Ecuador 2016”.

**Materials and Methods:**

The study was communicational, quantitative, descriptive and relational. The population consisted of 279 records from the study of the epidemiological map of schoolchildren in the “El Vecino” parish. The database included the registration number, age, sex, parish and DMFT Index.

**Results:**

There was an statistically significant inverse correlation between the number of brushes per day and the DMFT index with a p value = 0.029.

**Conclusion:**

The present study shows that there is a correlation between the number of brushes per day and the DMFT index.

## INTRODUCCIÓN

Las enfermedades bucodentales, como la caries dental, el cáncer de la boca, el cáncer de faringe y la periodontitis son un problema de salud de alcance mundial y cada vez más habitual, lo cual afecta en mayor escala a los países en proceso de desarrollo. En especial, se presentan entre las comunidades de más bajos recursos, ya que en ellas las personas no poseen un programa de salud adecuado a su alcance ^1^.

El cepillado de los dientes se considera un comportamiento de autocuidado fundamental para el mantenimiento de la salud bucal, y el cepillado dos veces al día se ha convertido en una norma social, pero la base de evidencia para esta frecuencia es débil ^2^.

Las enfermedades bucodentales no solo tienen consecuencias para la salud, sino que también repercuten en lo económico, social y psicológico. Pueden tener efectos intermedios o negativos, como dolor, incomodidad y riesgo funcional, la cual causa disgusto con la apariencia y las actividades que realiza cada persona ^3^.

La enfermedad crónica más común entre los niños de todo el mundo es la carie dental. Aunque no suele ser mortal, tiene un impacto negativo en el bienestar general de las personas, ya que impide llevar una vida normal ^4^.

El dolor es un síntoma común de la caries dental y puede ser insoportable, lo que llega a tener efectos negativos en la alimentación, el desarrollo del niño, el aumento de peso y puede causar anemia cuando se relaciona con la inflamación crónica. La mala alimentación, la falta de aseo y la ingesta no controlada de dulces pueden afectar las piezas dentales; por lo tanto, estas costumbres no solo crean trastornos sistémicos, sino que también se manifiestan a nivel bucal, a través de un gran incremento de biopelícula, la cual es la causante primordial de las enfermedades orales. Por ello, existe una correlación entre dieta, peso y caries ^4^.

Según datos publicados por la Organización Mundial de la Salud (OMS), se calcula que entre el 60% y el 90% de los escolares tienen caries dental. El índice de CPOD (promedio de piezas definitivas cariadas, perdidas u obturadas) en Ecuador por caries, a la edad de 6 a 7 años, es del 0,22%, porcentaje que crece al 2,95% a la edad de 12 años y al 4,64% (CPOD) a los 15 años. Esto representa un nivel severo, de acuerdo con lo establecido por la OPS/OMS ^5^.

En Cuenca, el índice CPOD poblacional en los escolares fue del 3,69% y no mostró diferencias significativas entre el sexo masculino y femenino, con valores entre el 3,89% y el 3,55% ^6^.

El número de mutaciones de estreptococos en la saliva de los niños depende del número de lesiones cariosas en las fosas y grietas. En este estudio usaremos el índice CPOD, propuesto por Klein, Palmer y Knutson en 1938 ^7^.

Para la prevención de la caries, la medida más segura es el uso de flúor, el cual evita la propagación bacteriana de ácidos en boca y detiene la desmineralización del esmalte dental. Por ello, lo recomendable es aplicar la técnica de cepillado con pasta dental fluorada, ya que se trata de uno de los procesos más costo-efectivos y eficaces para disminuir el riesgo de caries y prevenir enfermedades en la boca ^8^.

Como objetivo de esta investigación, vamos a establecer la relación entre números de cepillados por día y CPOD en escolares de 12 años de la parroquia El Vecino (Cuenca, Ecuador) en 2016.

## MATERIALES Y MÉTODOS

El estudio fue de tipo descriptivo, correlacional y retrospectivo. La población estuvo conformada por 279 fichas que pertenecen al estudio del mapa epidemiológico para los escolares de la parroquia El Vecino, base de datos de ficha de observación que incluye lo siguiente: número de registro, edad, sexo, parroquia e índice de CPOD.

Mediante la exploración clínica y el llenado del formulario OMS de evolución de la salud bucodental, se identificó las piezas que presentaban CPOD, el número de cepillados por día y el grado de severidad. Para llevar a cabo estos procesos se envió un oficio al jefe de distrito del cantón Cuenca, a fin de facilitar el ingreso a las instituciones educativas de diversas parroquias pertenecientes al cantón. En dicho oficio se indicó la necesidad de realizar un estudio con los escolares de 12 años y tener la aprobación correspondiente de los padres de familia.

En el presente estudio se analizará la VAR X mediante estadística descriptiva (tablas y gráficos) y luego se realizará el mismo procedimiento con la VAR Y. Una vez conocidas, se podrá garantizar que la información sea actualizada y verídica, como el sexo, el número de cepillados, entre otros.

La herramienta que se utilizó fue la Tau de Kendall, que nos permitió correlacionar la variable, el índice CPOD y el número de cepillados por día. Para la medición de la variable, se utilizó ficha de observación.

La presente investigación no implicó ningún conflicto bioético, debido a que fue un estudio retrospectivo. La información reposa en la oficina de investigación de la carrera de Odontología; además, se guardó la respectiva confidencialidad sobre los datos proporcionados.

## RESULTADOS

En el presente estudio, el total de la muestra es de 279 niños, de los cuales 171 (61,3%) eran del sexo femenino y 108 (38,7%), del masculino. Por otro lado, en referencia a los niveles de CPOD, encontramos que el sexo femenino presentó mayor porcentaje en el parámetro de muy alto, con un 28,1%, y menor porcentaje en el rango de bajo, con un 9,4%. Mientras que el sexo masculino mostró el mayor porcentaje en el rango muy alto, con un 29,6%, y un menor porcentaje, con un 12,0%, en los rangos de bajo y muy bajo. También se encontró que, en la correlación con el número de cepillados, el sexo femenino presenta el porcentaje más alto en el parámetro tres veces al día, con un 57,3%, y el sexo masculino muestra un 59,3% en el parámetro menos de tres veces al día (Tabla 1).

**Tabla 1:** Distribución de la muestra según sexo, niveles de CPOD y número de cepillados

SEXO			NIVELES DE CPOD			NÚMERO DE CEPILLADOS			
	n	%		s	%		N	%
FEMENINO	171	61,3	MUY BAJO	23	13,5	MENOS DE 3 VECES AL DIA	73	42,7
			BAJO	16	9,4	TRES VECES AL DIA	98	57,3
			MEDIO	42	24,6	MÁS DE 3 VECES AL DIA	0	0
			ALTO	42	24,6			
			MUY ALTO	48	28,1			
MASCULINO	108	38,7	MUY BAJO	13	12,0	MENOS DE 3 VECES AL DIA	64	59,3
			BAJO	13	12,0	TRES VECES AL DIA	43	39,8
			MEDIO	26	24,1	MÁS DE 3 VECES AL DIA	1	0,9
			ALTO	24	22,2			
			MUY ALTO	32	29,6			
TOTAL	279			279			279	

**Figura 1:**
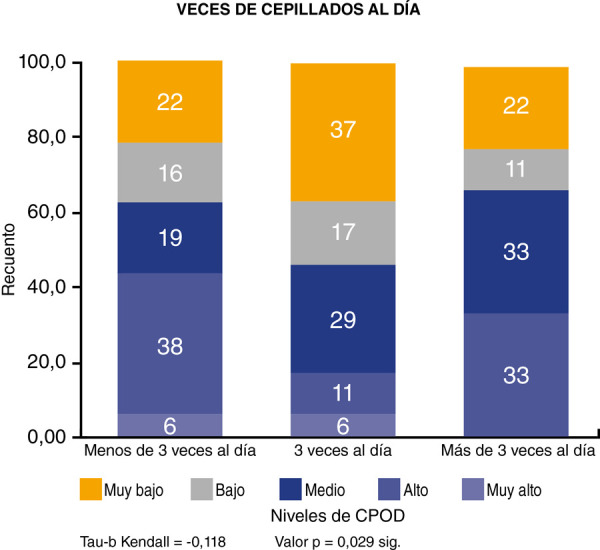
Correlación entre número de cepillados por día e índice de CPOD

**Figura 2:**
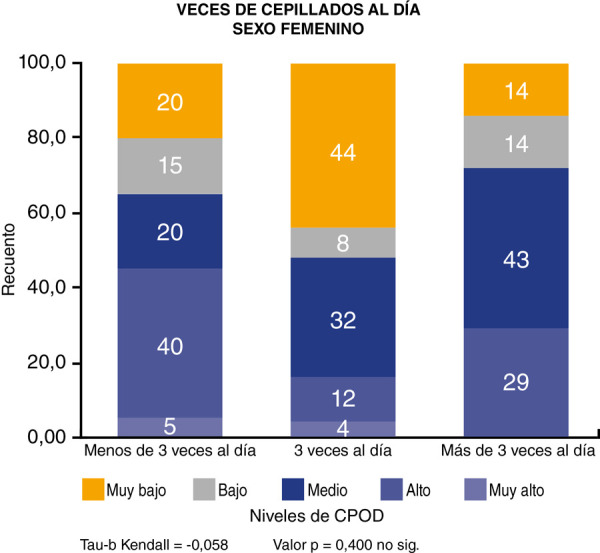
Correlación entre número de cepillados por día e índice de CPOD en el sexo femenino

**Figura 3:**
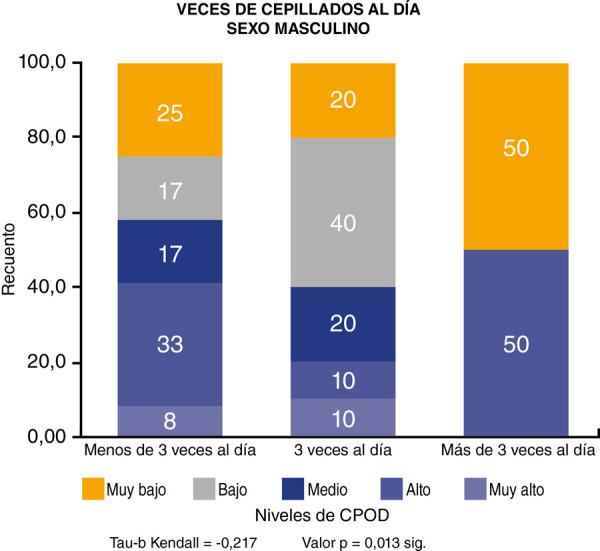
Correlación entre número de cepillados por día e índice de CPOD en el sexo masculino.

En lo referente a la correlación entre el número de cepillado por día y el índice de CPOD, se demostró que existe una correlación inversa, estadísticamente significativa, con un valor de p = 0,029 (Fig. 1). En el sexo femenino encontramos que la correlación es estadísticamente no significativa p = 0,400 (Fig. 2), mientras que en el sexo masculino la correlación es estadísticamente significativa p = 0,013 (Fig. 3).

## DISCUSIÓN

La caries en esta investigación se presentó en un nivel muy alto, con un porcentaje general del 28,7%, valor desfavorable si se considera que la prevalencia de caries a nivel de Latinoamérica va de muy baja a moderada, con un rango de 0,79 a 3,68 ^8^.

Si consideramos la caries y el sexo, se evidenció que presentan valores similares, con una diferencia del 1,5% en el sexo masculino y un nivel de CPOD en muy alto para ambos sexos. De la misma manera, un estudio realizado en la ciudad de México obtuvo un promedio de CPOD de 3,0 para el sexo femenino y 2,9 para el masculino, sin significancia estadística, pero se puede observar que la experiencia de caries en la población mexicana fue mayor ^9^.

Por otro lado, analizamos un artículo referido a la parroquia Llacao, dentro de la misma cuidad que nuestro estudio, en el cual observamos que el nivel de severidad en el sexo femenino fue del 34% y en el masculino, del 28%, lo que corresponde a niveles muy bajos de CPOD, con un promedio de 3,7 en ambos sexos, mientras que en nuestro estudio, en la parroquia El Vecino, se encontraron prevalencias muy altas, con el 28,7% de CPOD en ambos sexos ^13^.

En el presente estudio, la experiencia de caries fue del 28,1% en el sexo femenino y del 29,6% en el masculino, lo que demostró que los escolares de la parroquia El Vecino presentan un nivel muy alto de caries, resultados que fueron similares a los obtenidos por Padilla, quien refiere que el CPOD en el sexo masculino fue del 28,71% y en el sexo femenino, del 30,05%, sin encontrar estadística significativa p = 0,67 ^16^.

En lo referente al número de cepillados por día, este estudio arrojó que el 57,3% de los individuos del sexo femenino cepilla sus dientes tres veces al día y que el 59,3% de los individuos masculinos cepillan menos de tres veces al día sus dientes. Estudios a nivel mundial indican que la frecuencia de cepillados está en más de una vez al día. Lafuente, en su estudio aplicado a una población de adolescentes españoles, obtuvo que el 41,95% cepillan sus dientes más de tres veces al día y un 4,3% de estos no realiza ningún cepillado diario. Se evidencia que existe mayor prevalencia de este hábito en el sexo femenino, con un 54,1%, frente a un 29,3% en el caso del sexo masculino ^19^.

## CONCLUSIÓN

En esta investigación, la asociación de las variables número de cepillados por día e índice de CPOD demostró que existe una correlación inversa, estadísticamente significativa, con un valor p = 0,029. Una de las limitaciones de este trabajo fue la escasa literatura científica sobre estudios de correlación entre las variables anunciadas, lo que constituyó una limitante al momento de realizar el análisis de los resultados, por lo que se sugiere que el presente estudio sirva como base para futuras investigaciones.

Se considera necesario implementar planes y programas de prevención, como charlas sobre técnicas de cepillado dental, higiene bucal, y brindar información sobre cómo deberían ser las visitas al odontólogo y qué tan necesarias resultan para mantener una buena salud bucal y un buen estilo de vida.

## Fuente de financiamiento:

Autofinanciado
